# Discovery of serum biomarkers of alcoholic fatty liver in a rodent model: C-reactive protein

**DOI:** 10.1186/1423-0127-18-52

**Published:** 2011-08-01

**Authors:** Shu-Lin Liu, Chun-Chia Cheng, Chun-Chao Chang, Fu-Der Mai, Chia-Chi Wang, Shui-Cheng Lee, Ai-Sheng Ho, Ling-Yun Chen, Jungshan Chang

**Affiliations:** 1 Institute of Biochemistry and Biotechnology, Chung Shan Medical University, Taichung, Taiwan; 2 Graduate Institute of Medical Sciences, College of Medicine, Taipei Medical University, Taipei, Taiwan; 3 Institute of Nuclear Energy Research, Atomic Energy Council, Taoyuan, Taiwan; 4 Department of Internal Medicine, School of Medicine, College of Medicine, Taipei Medical University Hospital, Taipei, Taiwan; 5 Department of Biochemistry, School of Medicine, Taipei Medical University, Taipei, Taiwan; 6 Biomedical Mass Imaging Research Center, Taipei Medical University, Taipei, Taiwan; 7 Division of Gastroenterology, Buddhist Tzu Chi General Hospital, Taipei branch, Taiwan; 8 Division of Gastroenterology, Cheng Hsin General Hospital, Taipei, Taiwan; 9 Research Center For Biomedical Implants and Microsurgery Devices, Taipei Medical University, Taipei, Taiwan; 10 Neuroscience Research Center, Taipei Medical University Hospital, Taipei, Taiwan

**Keywords:** alcoholic fatty liver, biomarker, C - reactive protein, haptoglobin, two-dimensional differential gel electrophoresis

## Abstract

**Background:**

Excessive consumption of alcohol contributes to alcoholic liver disease. Fatty liver is the early stage of alcohol-related liver disease. The aim of this study was to search for specific serological biomarkers of alcoholic fatty liver (AFL) compared to healthy controls, non-alcoholic fatty liver (NAFL) and liver fibrosis in a rodent model.

**Methods:**

Serum samples derived from animals with AFL, NAFL, or liver fibrosis were characterized and compared using two-dimensional differential gel electrophoresis. A matrix-assisted laser desorption ionization-time of flight tandem mass spectrometer in conjunction with mascot software was used for protein identification. Subsequently, Western blotting and flexible multi-analyte profiling were used to measure the expressions of the putative biomarkers present in the serum of animals and clinical patients.

**Results:**

Eight differential putative biomarkers were identified, and the two most differentiated proteins, including upregulated C-reactive protein (CRP) and downregulated haptoglobin (Hp), were further investigated. Western blotting validated that CRP was dramatically higher in the serum of AFL compared to healthy controls and other animals with liver disease of NAFL or liver fibrosis (*p *< 0.05). Moreover, we found that CRP and Hp were both lower in liver fibrosis of TAA-induced rats and clinical hepatitis C virus-infected patients.

**Conclusion:**

The results suggest that increased levels of CRP are an early sign of AFL in rats. The abnormally elevated CRP induced by ethanol can be used as a biomarker to distinguish AFL from normal or otherwise diseased livers.

## Background

Excessive alcohol consumption affects lipid metabolism in the liver [1,2], contributing to the development of alcohol-related liver diseases. There are three main types of alcohol-related liver disease, these are: alcoholic fatty liver (AFL), alcoholic hepatitis, and alcoholic cirrhosis. AFL is the early stage of alcohol-related liver diseases. Therefore, identifying putative serum biomarkers of AFL for early and accurate diagnostic methods is vital.

Histological assessment of liver biopsy specimens remains the gold standard for determining alcohol-related liver disease. However, the methodology of histological assessments needs to overcome several drawbacks such as its invasive character and sampling error [3]. Moreover, it has difficulty in distinguishing AFL from non-alcoholic fatty liver solely through a histological assessment. On the other hand, predicting ethanol-induced oxidative stress and tissue injury in the liver require particularly sensitive markers [4]. A previous study suggested that glutamyl-transpeptidase (GGT) and alanine aminotransferase (ALT) are biomarkers for diagnosing alcoholic liver disease [5]. Several reports proposed that serum C-reactive protein (CRP), tissue polypeptide-specific antigen (TPS), and interleukin-6 are noninvasive biomarkers of alcoholic hepatitis [6-10]. Nevertheless, a reliable biomarker to predict the early stage of alcoholic hepatitis, i.e., AFL, and to distinguish AFL from other types of liver disease is needed.

A proteomics strategy based on two-dimensional differential gel electrophoresis (2D-DIGE) [11] allows for the simultaneous resolution of thousands of proteins from samples with high precision and replication. 2D-DIGE was used to screen and determine putative biomarkers of many diseases [12-15]. Those protein samples of interest displayed on 2D-DIGE can be extracted and acquired from gels for identification and further investigation. In total, we discovered eight differential proteins associated with AFL using 2D-DIGE, including the most differentiated proteins: CRP and haptoglobin (Hp).

This study revealed that CRP is a novel early biomarker of alcohol-induced fatty liver, and that CRP and Hp were both particularly decreased with liver fibrosis. In conclusion, we present CRP as a surveillance marker of alcohol-induced fatty liver in a rodent model, which may help diagnose early alcohol-induced pathophysiological alterations in clinical practice.

## Materials and methods

### Animal model and sample preparation

Animal experimentation was performed according to approved procedures of the Institute of Nuclear Energy Research, Atomic Energy Council, Taoyuan, Taiwan (approval no.: 98053). Wistar rats were used to generate animals with AFL, non-AFL (NAFL), and liver fibrosis. AFL rats (*n *= 6) were orally fed 5 ml of a 36% alcohol solution for 4 weeks (6 g/kg/day) [16]. For rats with NAFL, animals were given food containing 60% fructose (*n *= 4) or 45% fat (n = 6) for 12 weeks. The liver-fibrosis rats (*n *= 6) were fed 0.04% thioacetamide (TAA)-containing drinking water for 12 weeks. Control animals were fed normal diets with no additives in their food (*n *= 7). Sera and liver tissues were collected for further investigation.

### Clinical serum collection

The sera of healthy volunteers (*n *= 16), patients with non-alcoholic steatohepatitis (*n *= 19) and patients with hepatitis C virus (HCV)-infected liver fibrosis (*n *= 17) were obtained from Cheng Hsin General Hospital in Taiwan (approval no. 97016). A liver biopsy and subsequent histological examination were used to assess the stage of liver fibrosis according to the Metavir classification, and also to determine the fatty change and modified HAI grade. A liver biopsy was not performed in healthy controls due to ethical issues.

### 2D-DIGE

Each 50 μg of protein from a normal control or AFL rat was labeled with 400 pmol of Cy3 or Cy5 and the internal pooled standard (100 μg) was labeled with 800 pmol of Cy2 for 30 min. The three labeled samples were pooled together for analysis. IPG strips (18 cm) at pH 4~7 for the first-dimension IEF (Ettan IPGphor System, GE Healthcare) and 12.5% polyacrylamide gels for the second dimension were used to separate serum proteins. The Cy2, Cy3, and Cy5-labeled images were acquired on a Typhoon TRIO Variable Mode Imager (GE Healthcare) using 488-, 532-, and 633-nm lasers with respective emission filters of 520, 532, and 670 nm. Images were analyzed using DeCyder 6.5 software (GE Healthcare) to select the differential proteins. Protein spots of interest were selected according to an independent Student's t-test with a significant value of < 0.05.

### Protein identification

In-gel digestion and MALDI-TOF MS analysis were performed as previously described [13].

### Western blotting

Each serum sample was diluted 1: 1 with a sodium dodecylsulfate (SDS) buffer containing 50 mM of Tris-Cl, 8 M urea, 30% glycerol, 2% SDS, 20 mM of dithiothreitol, and 0.1% bromophenol blue. A 4%~12% SDS- polyacrylamide gel electrophoresis (PAGE) (Invitrogen) was performed to separate the proteins. The iblot (Invitrogen) was used to transfer proteins to a polyvinylidene difluoride (PVDF) membrane. After using 0.5% milk to blot the PVDF membrane for 30 min, CRP and Hp were detected by a mouse anti-CRP immunoglobulin G (IgG) (Affinity BioReagents) and a mouse anti- Hp IgG (Sigma) for at least 1 h respectively. The second antibody conjugated with horseradish peroxidase (HRP) was incubated for 1 h at room temperature. The membranes were washed three times in phosphate-buffered saline (PBS; 10 mM sodium phosphate (pH7.4) and 0.9% NaCl) between adding antibodies. The Imaging System (Gel Doc XR System, Bio-Rad) was used to acquire images depending on a moderate exploration time and to semi-quantify protein expressions.

### Measurement of CRP and Hp concentration

Flexible multi-analyte profiling (xMAP) was performed to measure serum concentrations of CRP and Hp in clinical samples using the commercial Bio-Plex Pro Human Acute Phase 4-Plex Panel (Bio-Rad). The measurment procedure followed instructions in the manual.

### Statistical analysis

The statistical software, SPSS, was used to calculate the significance according to Student's t-test. Significance (*p *value) was accepted as < 0.05.

## Results

### Animal models

Each experimental animal bearing a specific liver disease, namely: AFL, NAFL and, liver fibrosis was analyzed and compared. To ensure the correct establishment of the animal models, several indicators in the serum including aspartate aminotransferase (AST), alanine aminotransferase (ALT), total bilirubin (TBIL), total cholesterol (TCHO) and triglyceride (TG) were measured and compared (Table 1). Levels of AST, ALT, and TBIL increased in LF rats [199 ± 37 U/L (*p *< 0.01), 74.4 ± 19 U/L (*p *< 0.01), and 0.84 ± 0.10 mg/dl (*p *< 0.05), respectively] compared to those in normal controls (AST 154 ± 25 U/L; ALT 56 ± 15 U/L; and TBIL 0.70 ± 0.06 mg/dl), indicating that the liver function of LF rats was impaired. However, no significant changes in these three serum indicators in AFL and NAFL were observed. Other indicators, TCHO and TG, were measured to observe lipid accumulation in the liver. An increased TG level was observed only in the group of rats fed the high concentration of fructose (91 ± 14 mg/dl, *p *< 0.05) compared to other rats fed different diets, but the TCHO level remained unchanged. These results show that AST, ALT, and TBIL increased in the serum of LF rats, and TG increased in rats that were fed a high level fructose. Therefore, these existing serum indicators so far could not be used to distinguish AFL from the normal controls, which means that finding differential biomarkers of AFL is vital.

**Table 1:** Levels of some clinical serum indicators in the animals

Indicators	Normal (*n *= 7)	AFL (*n *= 6)	HF-NAFL (*n *= 4)	HL-NAFL (*n *= 6)	LF^§^ (*n *= 5)
BW (g)	270 ± 5^¶^/457 ± 14^§^	275 ± 3^¶^	418 ± 28^§^	565 ± 29^§,^*	ND
AST (U/L)	154 ± 25	148 ± 34^c^	164 ± 31	181 ± 63	199 ± 37*
ALT (U/L)	56 ± 15	41 ± 13^c^	48 ± 17	57 ± 22	74 ± 19*
TBIL (mg/dl)	0.70 ± 0.06	0.62 ± 0.09^a, b, c^	0.78 ± 0.04	0.75 ± 0.05	0.84 ± 0.10*
TG (mg/dl)	51 ± 16	58 ± 10^a^	91 ± 14**	56 ± 11	48 ± 14
TCHO (mg/dl)	61 ± 10	68 ± 8	72 ± 10	71 ± 12	61 ± 17

BW, body weight; AST, aspartate aminotransferase; ALT, alanine aminotransferase; TBIL, total bilirulin; TG, triglyceride; TCHO, total cholesterol; AFL, alcoholic fatty liver; HF-NAFL, high fructose-induced non-alcoholic fatty liver; HL-NAFL, high lipid-induced non-alcoholic fatty liver; LF, liver fibrosis. Indicators for predicting liver function include GOT, GPT, and bilirubin and for predicting fat cells include triglyceride and cholesterol. Rats were treated before the age of 6 weeks and sacrificed by the ages of ^¶^10 and ^§^18 weeks. ND, Non-detection. The *p *value was calculated according to Student's *t*-test. * *p *< 0.05 and **p < 0.01 as compared to normal controls. A significant change (*p *< 0.05) was also determined for AFL rats compared to ^a^HF-NAFL, ^b^HL-NAFL, and ^c^LF rats.

**Figure 1:**
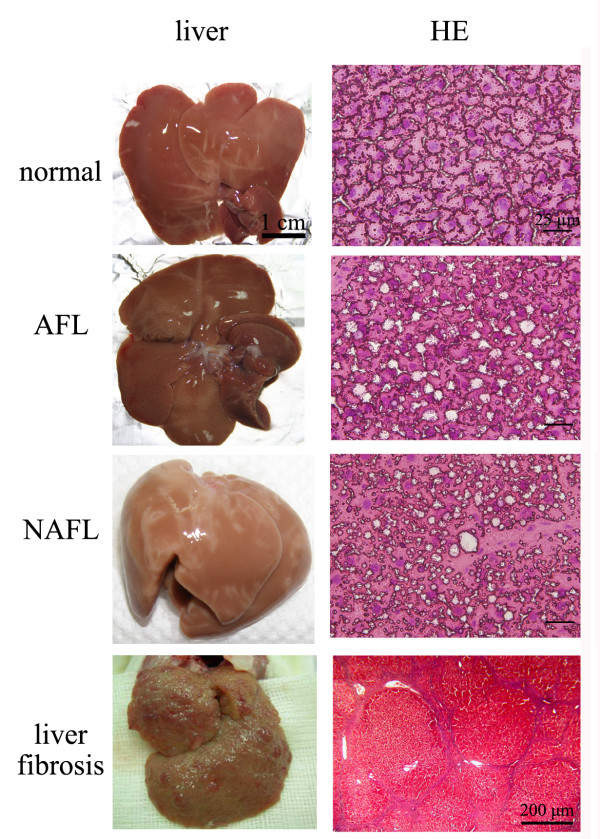
**Histological analysis and comparisons among four groups of rats**. Images of livers from normal control, rats with alcoholic fatty liver (AFL), non-alcoholic fatty liver (NAFL, fed 60% fructose) and liver fibrosis (left column) were analyzed by hematoxylin and eosin (H&E) staining (right column). In AFL and NAFL rats, livers were filled with prominent fatty change. The scale bar represents 25 μm for normal, AFL and NAFL; but 100 μm for liver fibrosis.

On the other hand, we observed that there was no significant difference in the morphology of livers among normal, AFL and, NAFL rats except for rats with liver fibrosis showing excess scars (Figure 1). To more-deeply assess our established rodent models using histological examinations, the results showed that livers of rats with AFL appeared to specifically be filled with macrovesicular fat within hepatocytes compared to normal controls according to histological staining with hematoxylin and eosin (H&E) (Figure 1), demonstrating that ethanol treatment induced lipid accumulation in the liver. Furthermore, signs of focal necroinflammation were absent from the liver tissues of rats with AFL (Figure 1).

### Discovery of AFL biomarkers using 2D-DIGE

**Figure 2:**
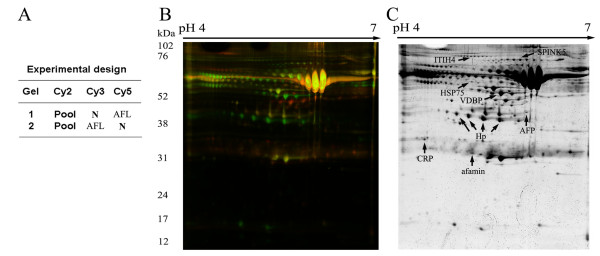
**Determination of serum biomarkers of alcoholic fatty liver (AFL) using 2D-DIGE**. (A) The experimental design used two individual samples each from healthy controls and AFL. (B) The combination of two different acquired images, in which, green color represents Cy3-labeled normal control and red color represents Cy5-labeled AFL. (C) The identified proteins are indicated by arrows on the 2D gel. Ultimately there were eight proteins selected according to statistical significance with a *t*-test value of < 0.05 (*p *< 0.05) as analyzed by the DeCyder software. Vitamin D-binding protein (VDBP), haptoglobin (HP), C-reactive protein (CRP), alpha-fetoprotein (AFP), inter-alpha-inhibitor H4 heavy chain (ITIH4), serine protease inhibitor Kazal-type 5 (SPINK5) and heak shock protein 75 kDa (HSP75) and afamin.

**Figure 3:**
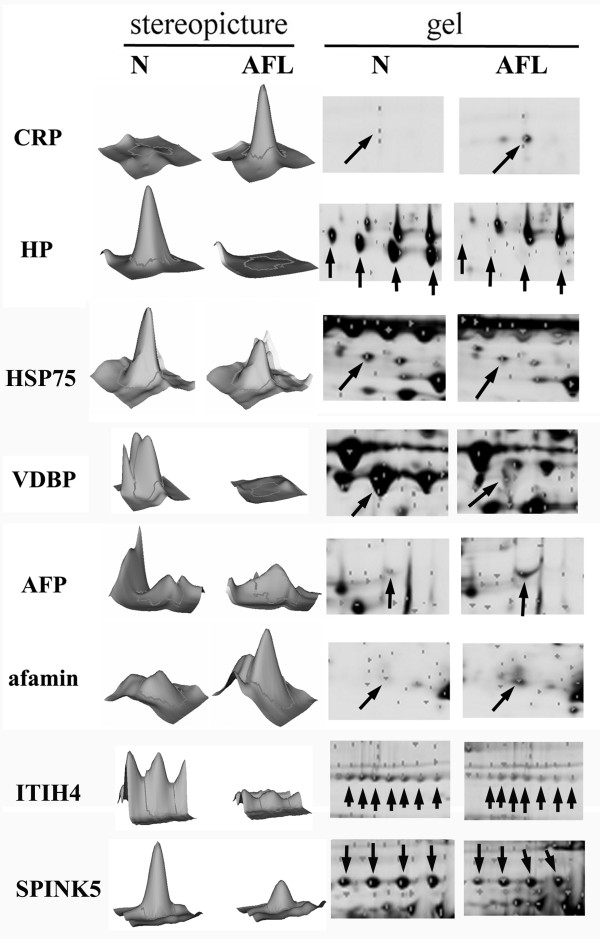
**Stereopictures and detailed images of eight putative biomarkers**. VDBP, vitamin D binding protein; HP, haptoglobin; CRP, C-reactive protein; AFP, alpha-fetoprotein; ITIH4, Inter-alpha-inhibitor H4 heavy chain; SPINK5, Serine protease inhibitor Kazal-type 5; HSP75, Heak shock protein 75 kDa. The protein spots are indicated by arrows. Three proteins, CRP, AFP and afamin, were upregulated whereas the other proteins, including Hp, ITIH4, APINK5, HSP75 and VDBP, were downregulated.

In order to explore the signature molecular biomarkers of AFL, a proteomic methodology, 2D-DIGE, described above "Materials and methods" was performed to analyze individual serum from two normal controls or two AFL rats shown on Figure 2A to search for putative biomarkers of AFL. In the 2D-DIGE analysis, Cy3 and Cy5 were used to individually label serum samples from normal controls and AFL rats. For sample normalization, Cy2 was used to label the internal standard including 50% of normal and 50% of AFL rats. The protein image was presented as shown in Figure 2B. Normal control sera were labeled with Cy3 and appeared colored green in the gel. Samples derived from rats with AFL were pre-labeled with Cy5 and showed as a red color in the gel. Eight differential proteins including CRP, Hp, afamin, alpha-fetoprotein (AFP), inter-alpha-inhibitor H4 heavy chain (ITIH4), serine protease inhibitor Kazal-type 5 (SPINK5), heak shock protein 75 kDa (HSP75), and vitamin D binding protein prepeptide (VDBP) were acquired according to the statistical analysis with significant *p *values (*t*-test, *p *< 0.05), and an intensity change ratio of > 1.2-fold calculated with DeCyder software. The location of each protein is shown in Figure 2C. When using a stereopicture and detailed gel images to present the protein expressions of differential biomarkers (Figure 3), CRP, AFP and afamin were increased higher in the serum of AFL rats, and Hp, ITIH4, SPINK5, HSP75, and VDBP were conversely lower. In particular, Hp, ITIH4 and SPINK5 had a series of spots nearby, but the protein expressional trends were still the same. We speculated that post-translational modification would not affect or influence the protein expression. Essentially, we identified protein spots by comparing the mass spectrum obtained from MALDI-TOF MS coupled with the NCBI database. The proteins identified are shown in Table 2. According to the set calculation in the DeCyder software, the upregulation (+) is presented as the levels of AFL divided by that of normal control, and the downregulation (-) is presented as the levels of normal control divided by that of AFL. The results demonstrated that CRP and Hp were dramatically up- and downregulated, respectively (CRP: +4.71-fold; HP: -11.54-fold, Table 2).

**Table 2:** Protein spots identified by MALDI-TOF/TOF MS

Gene name	Protein name	Mr (Da)/pI	Coverage ratio	Regulation^¶^	*p *value
Crp	C-reactive protein	25452/4.89	7%^§^	+4.71	0.02
Afm	Afamin	49311/6.14	59%	+1.64	0.04
Afp	Alpha-fetoprotein	47195/5.47	3%^§^	+1.46	0.03
Hp	Haptoglobin	39052/6.10	48%	-11.54	0.04
Gc	Vitamin D-binding protein	53493/5.65	4%^§^	-1.74	0.04
Itih4	Inter-alpha-inhibitor H4 heavy chain	103885/6.08	48%	-1.66	0.03
Spink5	Serine protease inhibitor Kazal-type 5	114816/8.68	74%	-1.67	0.04
Trap1	Heak shock protein 75 kDa, mitochondrial	80639/6.56	57%	-1.96	0.02

^§^Those proteins were identified by MS/MS. ^¶ ^Presented as up- (+) or down regulation (-) compared to normal controls.

### CRP and Hp validation

**Figure 4:**
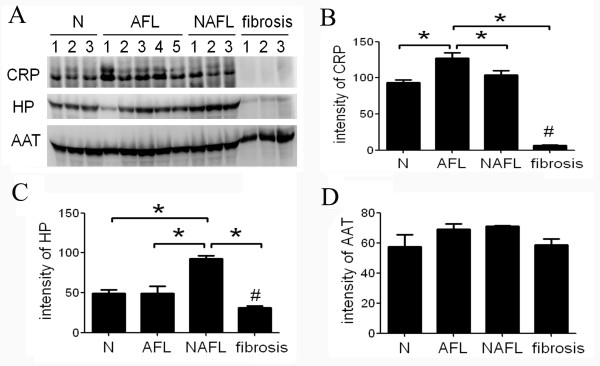
**Evaluation of the expressions of alcoholic fatty liver (AFL) biomarkers using western blotting**. (A) Confirmation of C-reactive protein (CRP) and haptoglobin (Hp) expression by Western blotting. AAT was used as an indicator represented as a positive inflammatory protein for gastrointestinal inflammation (B) The semi-quantification of the results of Western blotting. CRP increased in the serum of AFL rats compared to normal, NAFL and liver fibrosis ones, but decreased in TAA-induced liver fibrosis. (C) Hp increased in rats suffering from NAFL, but decreased in liver fibrosis compared to the controls. (D) AAT was not affected among the rats. Three individual samples of healthy controls, NAFL, and liver fibrosis, and five samples of AFL were examined by using Western blotting. N, normal controls; AFL, alcoholic fatty liver; NAFL, non-alcoholic fatty liver; CRP, C-reactive protein; Hp, haptoglobin; AAT, alph1 antitrypsin. *p < 0.05, compared to healthy controls; ^#^p < 0.05 as compared to the other groups.

We were interested in characterizing the role of CRP and Hp in AFL due to significant changes in their protein expressions. In the process of Western blotting, we precisely controlled the loading protein to 20 μg, and the total protein stained by SYPRO Ruby was used as a loading standard (data not shown). Figure 4A and 4B show that CRP particularly increased in AFL rats compared to all other groups including normal rats and rats with NAFL disease or liver fibrosis (all *p *< 0.05), demonstrating that CRP is a putative biomarker of AFL. Meanwhile, Hp did not significantly decrease in the serum of AFL rats according to the Western blotting analysis (Figure 4A). Interestingly, we observed that CRP and Hp were both downregulated in the serum of liver fibrosis rats compared to normal, AFL, and NAFL rats (Figure 4B, C, all *p *< 0.05). On the other hand, we also examined the level of alpha1 antitrypsin (AAT) to exclude the elevation of CRP derived from ethanol-induced gastrointestinal inflammation. The results showed that AAT levels were not increased in the serum of AFL rats compared to healthy controls (Figure 4D).

Moreover, in order to evaluate the decreased expressions of CRP and Hp in the serum of liver fibrosis rats, we measured serum CRP and Hp concentrations in clinical patients with non-alcoholic steatohepatitis (NASH) and HCV-induced liver fibrosis compared to healthy controls. Table 3 shows that CRP and Hp were lower in patients with HCV-induced liver fibrosis, which was consistent with the results demonstrated by Western blotting, suggesting that CRP and Hp are reliable biomarkers of liver fibrosis as downregulated proteins. Interestingly, we found that serum Hp was elevated in NAFL rats, but lower in NASH patients, implying that the expression of Hp may vary between the non-necroinflammatory stage (NAFL) and necroinflammatory stage (NASH).

**Table 3:** Serum concentration of C reactive protein (CRP) and haptoglobin (Hp) in clinical patients

Patients (*n*)	Fibrosis (*n*, %)	P-steatosis (*n*, %)	HAI (*n*, %)	CRP (mg/L)	Hp (g/L)
Healthy (16)	ND	ND	ND	1.58 ± 0.48	1.13 ± 0.63
NASH (19)	F1~F2 (19, 100%)	Stage 0~1 (7, 37%)	Stage 0~4 (18, 95%)	ND	0.74 ± 0.39*
		Stage 2~3 (12, 63%)	Stage 5~13 (1, 5%)		
HCV- liver fibrosis (17)	F1~F2 (7, 41%)	Stage 0~1 (15, 88%)	Stage 0~4 (2, 12%)	1.31 ± 0.44*	0.45 ± 0.58*^,#^
	F3~F4 (10, 59%)	Stage 2~3 (2, 12%)	Stage 5~13 (15, 88%)		

The fibrotic stage was determined according to the Metavir classification. The stage of p-steatosis was determined according to the fatty change. HAI, necroinflammatory scores. NASH, non-alcoholic steatohepatitis; ND, not determined.* *p *< 0.05, compared to healthy controls. ^# ^*p *< 0.05, compared to NASH. The significance was calculated according to Student's *t*-test. The data are presented as the mean ± SEM.

## Discussion

The aim of this study was to determine the putative serological biomarkers of AFL using 2D-DIGE, and then these candidate markers were validated by Western blotting. To characterize AFL disease markers, our experimental strategy was first to induce patholophysiological abnormalities in animals by administering alcohol, high calories of compounds such as: fructose or fats, and drinking water with TAA, a fibrosis-inducing chemical. Under this experimental platform, rats should have developed AFL, NAFL and liver fibrosis, allowing us to determine the signature biomarkers for AFL, which is the early stage in the alcohol-induced liver disease. From our rodent models, we determined that CRP levels were significantly elevated in the serum of rats with AFL, presumably as a reliable biomarker compared to that in livers of healthy or sick rats with other liver diseases.

Indeed, CRP increases in other conditions, such as inflammation, obesity [17,18], and cardiovascular disease [19,20]; however, it is also a elevated AFL-induced protein in rats as discovered in this study. Here, our discovery provides a hint that CRP can be used to distinguish AFL from normal or otherwise diseased livers. A previous report indicated that CRP is a non-invasive marker of alcoholic hepatitis in heavy drinkers compared to hepatitis unrelated to alcohol [7]. In this study, we discovered that CRP levels were elevated in AFL rats compared to healthy animals, and rats with other forms of liver diseases, such as NAFL and TAA-induced liver fibrosis. Although the use of moderate alcohol consumption can lower the level of CRP in the serum and decrease cardiovascular mortality [21], in our study the intake of high amounts of alcohol in this rodent model increased the level of serum CRP, suggesting that the intake of ethanol is positively associated with the level of serum CRP. To our knowledge, CRP is considered an inflammatory protein produced from macrophages in the liver and adipocytes [22,23]. In order to exclude elevated CRP derived from a response to gastrointestinal inflammation because of ethanol consumption, we examined protein levels of alpha1 antitrypsin, an acute-phase marker [24,25], determined by Western blotting, which provided a positive reference of an inflammatory response. The results showed that levels of alpha1 antitrypsin in rats among the four groups were the same (Figure 4A, D), indicating that the elevation of CRP was not due to gastrointestinal inflammation. Moreover, in vitro study revealed that ethanol can directly trigger the secretion of CRP in HepG2 cells (data not shown). Herein, the results suggest that ethanol-induced formation of fatty liver was strongly related to the induction of serum CRP in rats supplied with excess ethanol.

An increase in CRP was also reported to be associated with obesity [17,18]. To address our concern for the obesity issue, we also measured animal weight in all groups. We observed that macrovesicular fat was apparent in the liver tissues of rats fed diets containing a high fructose content (HF-NAFL) which did not increase serum CRP. In AFL rats, macrovesicular fat was also observed, but serum CRP was elevated. Therefore, this suggests that alcohol abuse may cause fatty liver and induce high serum CRP levels, indicating that CRP may be qualified as a unique biomarker of AFL.

Administration of ethanol can elicit oxidative stress and injury to the liver [4,26], and the presence of polyunsaturated fats can induce the production of cytochrome P4502E1 (CYP2E1) [27,28]. Under conditions of persistent ethanol stimulation, CYP2E1 seems to play a critical role in metabolizing and activating many toxicological substances such as reactive oxygen species (ROS) [26,29]. A recent study indicated that cytokines such as tumor necrosis factor-alpha and interleukin-10 in adipose tissues of acute alcoholic hepatitis patients were elevated, and were correlated with the serum CRP concentration [30], implying that inflammation caused the production of CRP. Although the mechanism of how ethanol induces CRP in serum is unclear, in this study, we discovered that ethanol consumption is an important factor positively associated with the production of serum CRP.

In addition to the increased CRP levels in AFL rats, serum Hp was also discovered to be a biomarker of AFL with reduced expression levels in the serum of AFL rats using 2D-DIGE. However, Hp was elevated in the serum of NAFL rats and even decreased in that of rats with TAA-induced liver fibrosis (Figure 4C). Previous studies reported by Chiellini's group indicated that elevated serum Hp is recognized as a marker of adiposity [31]. Our results demonstrate that Hp is not only increased in NAFL, but also decreased in TAA-induced and HCV-induced liver fibrosis. Furthermore, Shu et al. indicated that Hp was overexpressed in hepatocellular carcinoma compared to those with hepatitis B virus (HBV)-related cirrhosis [32]. However another research group led by Dr. Lee reported that Hp levels were decreased independently in hepatic fibrosis in chronic liver disease [33]. Therefore, the level of serum Hp may vary under various pathophysiological situations or stages in clinical liver diseases. Hp is also used in the panel of AshTest [34] as a down-regulated protein. In this study, we found that Hp was a downregulated protein in NASH and HCV-infected liver fibrosis although we found that Hp may be higher in the serum of NAFL rats. The results demonstrated that Hp is a reliable downregulated biomarker of NASH and liver fibrosis in clinical cases.

## Conclusions

For a diagnosis of alcoholic liver disease, a biopsy is the gold standard. Current studies focusing on the discovery of a noninvasive biomarker panel for diagnosis or prognosis imply that development of a noninvasive method is urgent. CRP is considered to be a marker of atherosclerotic cardiovascular disease in clinical analysis [19,20], modulating endothelial function in the process of atherogenesis [35]; therefore, we suggest that using CRP to distinguish AFL from the other liver diseases may consider the complication of cardiovascular disease. In conclusion, this is the first report to reveal new candidate biomarkers of AFL using a proteomics analysis. In this study, eight AFL-associated serological proteins were disclosed, which may be associated with AFL in rats. We suggest that CRP is suitable to serve as a candidate biomarker of AFL, and Hp is a reliable biomarker that decrease in NASH and liver fibrosis. In particular, serum CRP may be qualified to be an elevated surveillance target for early diagnosis of AFL in clinical screening.

## Competing interests

The authors declare that they have no competing interests.

## Authors' contributions

SLL: revised the article and designed the experiments. CCC: participated in article writing and performed most experiments, including 2D-DIGE, MALDI-TOF/TOF MS, and Western blotting. FDM and JS: Project leaders and corresponding authors, participated in this project in revising the article and providing opinions and interpretation of the data. SCL: provided suggestions and analysis regarding experimental outcomes as well as revised the article. CCW and CCC: performed tissue sampling and diagnosis. ASH: participated in the collection and diagnosis of clinical samples. LYC: Lab leader and article final revision, contributed to interpretation of the data. All authors read and approved the final manuscript.

## References

[B1] Rouach H Fataccioli V Gentil M French SW Morimoto M Nordmann R Effect of chronic ethanol feeding on lipid peroxidation and protein oxidation in relation to liver pathology Hepatology 1997 25 351 355 10.1002/hep.510250216 9021946

[B2] Grunnet N Kondrup J Dich J Effect of ethanol on lipid metabolism in cultured hepatocytes Biochem J 1985 228 673 681 3927897 10.1042/bj2280673 PMC1145037

[B3] Maharaj B Maharaj RJ Leary WP Cooppan RM Naran AD Pirie D Pudifin DJ Sampling variability and its influence on the diagnostic yield of percutaneous needle biopsy of the liver Lancet 1986 1 523 525 2869260 10.1016/s0140-6736(86)90883-4

[B4] Dey A Cederbaum AI Alcohol and oxidative liver injury Hepatology 2006 43 S63 74 10.1002/hep.20957 16447273

[B5] Levitsky J Mailliard ME Diagnosis and therapy of alcoholic liver disease Semin Liver Dis 2004 24 233 247 10.1055/s-2004-832937 15349802

[B6] Fujimoto M Uemura M Kojima H Ishii Y Ann T Sakurai S Okuda K Noguchi R Adachi S Kitano H Prognostic factors in severe alcoholic liver injury. Nara Liver Study Group Alcohol Clin Exp Res 1999 23 33S 38S 10.1111/j.1530-0277.1999.tb04531.x 10235276

[B7] Vanbiervliet G Le Breton F Rosenthal-Allieri MA Gelsi E Marine-Barjoan E Anty R Piche T Benzaken S Saint-Paul MC Huet PM Tran A Serum C-reactive protein: a non-invasive marker of alcoholic hepatitis Scand J Gastroenterol 2006 41 1473 1479 10.1080/00365520600842195 17101579

[B8] Gupta S Slaughter S Akriviadis EA Valenzuela R Deodhar SD Serial measurement of serum C-reactive protein facilitates evaluation in alcoholic hepatitis Hepatogastroenterology 1995 42 516 521 8751208

[B9] Gonzalez-Quintela A Mella C Perez LF Abdulkader I Caparrini AM Lojo S Increased serum tissue polypeptide specific antigen (TPS) in alcoholics: a possible marker of alcoholic hepatitis Alcohol Clin Exp Res 2000 24 1222 1226 10.1111/j.1530-0277.2000.tb02087.x 10968661

[B10] Hill DB Marsano L Cohen D Allen J Shedlofsky S McClain CJ Increased plasma interleukin-6 concentrations in alcoholic hepatitis J Lab Clin Med 1992 119 547 552 1583411

[B11] Unlu M Morgan ME Minden JS Difference gel electrophoresis: a single gel method for detecting changes in protein extracts Electrophoresis 1997 18 2071 2077 10.1002/elps.1150181133 9420172

[B12] Byrne JC Downes MR O'Donoghue N O'Keane C O'Neill A Fan Y Fitzpatrick JM Dunn M Watson RW 2D-DIGE as a strategy to identify serum markers for the progression of prostate cancer J Proteome Res 2009 8 942 957 10.1021/pr800570s 19093873

[B13] Ho AS Cheng CC Lee SC Liu ML Lee JY Wang WM Wang CC Novel biomarkers predict liver fibrosis in hepatitis C patients: alpha 2 macroglobulin, vitamin D binding protein and apolipoprotein AI J Biomed Sci 2010 17 58 10.1186/1423-0127-17-58 20630109 PMC2914022

[B14] Kondo T Hirohashi S Application of 2D-DIGE in cancer proteomics toward personalized medicine Methods Mol Biol 2009 577 135 154 10.1007/978-1-60761-232-2_11 19718514

[B15] Orenes-Pinero E Corton M Gonzalez-Peramato P Algaba F Casal I Serrano A Sanchez-Carbayo M Searching urinary tumor markers for bladder cancer using a two-dimensional differential gel electrophoresis (2D-DIGE) approach J Proteome Res 2007 6 4440 4448 10.1021/pr070368w 17902641

[B16] Keshavarzian A Farhadi A Forsyth CB Rangan J Jakate S Shaikh M Banan A Fields JZ Evidence that chronic alcohol exposure promotes intestinal oxidative stress, intestinal hyperpermeability and endotoxemia prior to development of alcoholic steatohepatitis in rats J Hepatol 2009 50 538 547 19155080 10.1016/j.jhep.2008.10.028 PMC2680133

[B17] Gentile M Panico S Rubba F Mattiello A Chiodini P Jossa F Marotta G Pauciullo P Rubba P Obesity, overweight, and weight gain over adult life are main determinants of elevated hs-CRP in a cohort of Mediterranean women Eur J Clin Nutr 2010 64 873 878 10.1038/ejcn.2010.69 20517327

[B18] Oda E CRP may be superior to anthropometric markers of obesity Circ J 2007 71 1332 author reply 1332-1333 10.1253/circj.71.1332 17652907

[B19] Martinez VB Gonzalez-Juanatey JR Markers of inflammation and cardiovascular disease: clinical applications of C-reactive protein determination Am J Cardiovasc Drugs 2009 9 Suppl 1 3 7 20000881 10.2165/1153161-S0-000000000-00000

[B20] Ridker PM Clinical application of C-reactive protein for cardiovascular disease detection and prevention Circulation 2003 107 363 369 10.1161/01.CIR.0000053730.47739.3C 12551853

[B21] Albert MA Glynn RJ Ridker PM Alcohol consumption and plasma concentration of C-reactive protein Circulation 2003 107 443 447 10.1161/01.CIR.0000045669.16499.EC 12551869

[B22] Pepys MB Hirschfield GM C-reactive protein: a critical update J Clin Invest 2003 111 1805 1812 12813013 10.1172/JCI18921 PMC161431

[B23] Das T Sen AK Kempf T Pramanik SR Mandal C Mandal C Induction of glycosylation in human C-reactive protein under different pathological conditions Biochem J 2003 373 345 355 10.1042/BJ20021701 12693993 PMC1223501

[B24] Laskowska-Klita T Czerwinska B [Concentration of C-reactive protein, procalcitonin and alpha-1-antitrypsin in blood of neonates and infants with signs of inflammation] Med Wieku Rozwoj 2002 6 5 11 12177508

[B25] Borawski J Naumnik B Mysliwiec M Serum alpha1-antitrypsin but not complement C3 and C4 predicts chronic inflammation in hemodialysis patients Ren Fail 2003 25 589 593 10.1081/JDI-120022550 12911163

[B26] Cederbaum AI Lu Y Wu D Role of oxidative stress in alcohol-induced liver injury Arch Toxicol 2009 83 519 548 10.1007/s00204-009-0432-0 19448996

[B27] Nanji AA Zhao S Lamb RG Dannenberg AJ Sadrzadeh SM Waxman DJ Changes in cytochromes P-450, 2E1, 2B1, and 4A, and phospholipases A and C in the intragastric feeding rat model for alcoholic liver disease: relationship to dietary fats and pathologic liver injury Alcohol Clin Exp Res 1994 18 902 908 10.1111/j.1530-0277.1994.tb00058.x 7978103

[B28] Cao Q Mak KM Lieber CS Cytochrome P4502E1 primes macrophages to increase TNF-alpha production in response to lipopolysaccharide Am J Physiol Gastrointest Liver Physiol 2005 289 G95 107 10.1152/ajpgi.00383.2004 15961886

[B29] Lu Y Cederbaum AI CYP2E1 and oxidative liver injury by alcohol Free Radic Biol Med 2008 44 723 738 10.1016/j.freeradbiomed.2007.11.004 18078827 PMC2268632

[B30] Naveau S Cassard-Doulcier AM Njike-Nakseu M Bouchet-Delbos L Barri-Ova N Boujedidi H Dauvois B Balian A Maitre S Prevot S Harmful effect of adipose tissue on liver lesions in patients with alcoholic liver disease J Hepatol 2010 52 895 902 10.1016/j.jhep.2010.01.029 20399524

[B31] Chiellini C Santini F Marsili A Berti P Bertacca A Pelosini C Scartabelli G Pardini E Lopez-Soriano J Centoni R Serum haptoglobin: a novel marker of adiposity in humans J Clin Endocrinol Metab 2004 89 2678 2683 10.1210/jc.2003-031965 15181041

[B32] Shu H Kang X Guo K Li S Li M Sun L Gan L Liu Y Qin X Diagnostic value of serum haptoglobin protein as hepatocellular carcinoma candidate marker complementary to alpha fetoprotein Oncol Rep 2010 24 1271 1276 20878120 10.3892/or_00000982

[B33] Lee HH Seo YS Um SH Won NH Yoo H Jung ES Kwon YD Park S Keum B Kim YS Usefulness of non-invasive markers for predicting significant fibrosis in patients with chronic liver disease J Korean Med Sci 2010 25 67 74 10.3346/jkms.2010.25.1.67 20052350 PMC2800033

[B34] Thabut D Naveau S Charlotte F Massard J Ratziu V Imbert-Bismut F Cazals-Hatem D Abella A Messous D Beuzen F The diagnostic value of biomarkers (AshTest) for the prediction of alcoholic steato-hepatitis in patients with chronic alcoholic liver disease J Hepatol 2006 44 1175 1185 10.1016/j.jhep.2006.02.010 16580087

[B35] Pasceri V Willerson JT Yeh ET Direct proinflammatory effect of C-reactive protein on human endothelial cells Circulation 2000 102 2165 2168 11056086 10.1161/01.cir.102.18.2165

